# A novel nomogram predicting the early recurrence of hepatocellular carcinoma patients after R0 resection

**DOI:** 10.3389/fonc.2023.1133807

**Published:** 2023-03-17

**Authors:** Huanhuan Wang, Runkun Liu, Huanye Mo, Runtian Li, Jie Lian, Qingguang Liu, Shaoshan Han

**Affiliations:** ^1^ Department of Hepatobiliary Surgery, The First Affiliated Hospital of Xi’an Jiaotong University, Xi’an, China; ^2^ Department of Pathology, The First Affiliated Hospital of Xi’an Jiaotong University, Xi’an, China

**Keywords:** hepatocellular carcinoma, early recurrence, nomogram model, MVI, VEGF-A

## Abstract

**Background:**

Early tumor recurrence is one of the most significant poor prognostic factors for patients with HCC after R0 resection. The aim of this study is to identify risk factors of early recurrence, in addition, to develop a nomogram model predicting early recurrence of HCC patients.

**Methods:**

A total of 481 HCC patients after R0 resection were enrolled and divided into a training cohort (n = 337) and a validation cohort (n = 144). Risk factors for early recurrence were determined based on Cox regression analysis in the training cohort. A nomogram incorporating independent risk predictors was established and validated.

**Results:**

Early recurrence occurred in 37.8% of the 481 patients who underwent curative liver resection of HCC. AFP ≥ 400 ng/mL (HR: 1.662; P = 0.008), VEGF-A among 127.8 to 240.3 pg/mL (HR: 1.781, P = 0.012), VEGF-A > 240.3 pg/mL (HR: 2.552, P < 0.001), M1 subgroup of MVI (HR: 2.221, P = 0.002), M2 subgroup of MVI (HR: 3.120, P < 0.001), intratumor necrosis (HR: 1.666, P = 0.011), surgical margin among 5.0 to 10.0 mm (HR: 1.601, P = 0.043) and surgical margin < 5.0 mm (HR: 1.790, P = 0.012) were found to be independent risk factors for recurrence-free survival in the training cohort and were used for constructing the nomogram. The nomogram indicated good predictive performance with an AUC of 0.781 (95% CI: 0.729-0.832) and 0.808 (95% CI: 0.731-0.886) in the training and validation cohorts, respectively.

**Conclusions:**

Elevated serum concentrations of AFP and VEGF-A, microvascular invasion, intratumor necrosis, surgical margin were independent risk factors of early intrahepatic recurrence. A reliable nomogram model which incorporated blood biomarkers and pathological variables was established and validated. The nomogram achieved desirable effectiveness in predicting early recurrence in HCC patients.

## 1 Introduction

Hepatocellular carcinoma (HCC) is the sixth most common cancer and the third leading cause of cancer death worldwide in 2020 (1). For patients with early-stage tumor (Barcelona Clinic Liver Cancer [BCLC] 0/A), surgical resection represents the most effective treatment, with curative potential (2). However, up to 68% of HCC patients might suffer from tumor recurrence within 5 years after surgery, even in patients with a single tumor of sizes ≤ 2 cm (3). Recurrence that occurs within two years after resection is defined as early recurrence (4). The median survival time for HCC patients with early recurrence is 15.8 months (5). Early recurrence of HCC after hepatic resection is predominantly responsible for dismal outcomes and is the major obstacle to improving HCC patients’ survival (6). Accurate risk prediction allows optimal surveillance, prevention, and management strategies for tumor recurrence. Therefore, it is urgent to identify effective predictors of early recurrence in HCC patients after liver resection.

Previous studies have shown that early recurrence is commonly associated with aggressive tumor pathological characteristics, such as large tumor size, poor cell differentiation, microvascular invasion (MVI), satellite nodules, and the absence of tumor capsule (7–10). MVI-positive patients are especially prone to develop an early recurrence after liver resection (11, 12). Increasing tumor stage, portal vein tumor thrombus (PVTT), hepatitis B surface antigen (HBsAg), hepatitis B virus-DNA (HBV-DNA) copy number and elevated alpha-fetoprotein (AFP) level have been reported to be associated with early recurrence (13, 14). However, most of these studies tend to explore the factors associated with increased risk of tumor recurrence, rather than enter the risk factors into multi-predictor models that consider their joint effects. A rapidly emerging field called radiomics has allowed digitally encrypted medical images to be transformed into innumerable quantitative features that provide information on tumor pathophysiology (15). Recently, radiomics-based models have shown the favorable predictive value of HCC recurrence (16, 17). One of the major limitations of radiomics-based models is that the computer-based algorithms in oncology to date have not been vigorously validated for reproducibility and generalizability (18). A reliable and applicable model for predicting early recurrence is still a pressing need. Serum vascular endothelial growth factor A (VEGF-A) levels are elevated in HCC patients (19), which are closely related to unfavorable prognosis in HCC patients (20). VEGF-A is associated with MVI as shown in previous studies (21, 22), which implies that VEGF-A may be associated with tumor recurrence.

In the present study, we conducted a retrospective cohort study of HCC patients underwent surgical resection to identify predictors of early recurrence. In addition, we developed a novel nomogram model incorporating AFP, VEGF-A, microvascular invasion, intratumor necrosis, and surgical margin, which can be used to accurately predict the risk of HCC recurrence.

## 2 Methods

### 2.1 Study population

**Figure 1:**
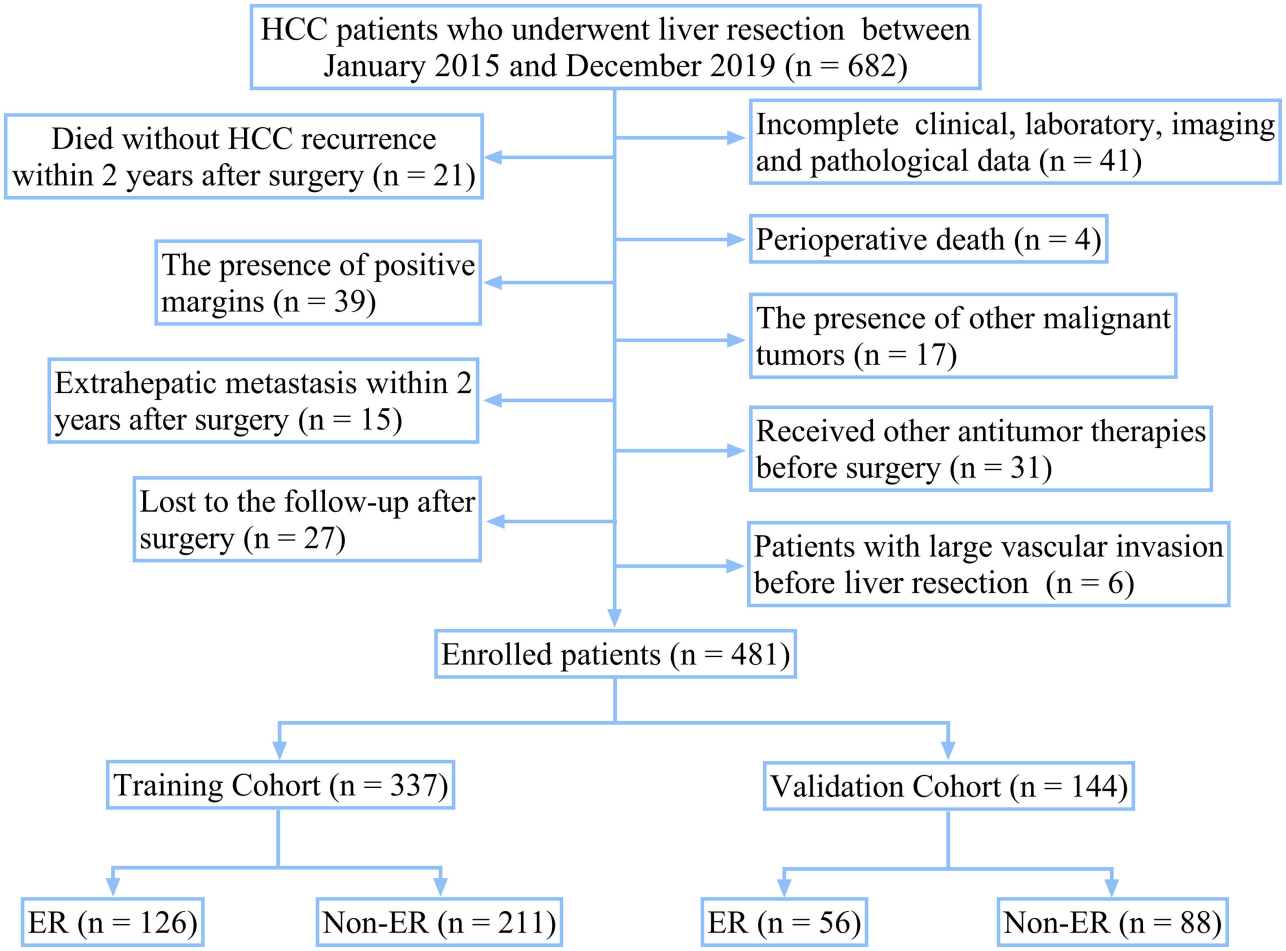
Flow chart for selecting eligible patients.

The selection procedure and study design are shown in **Figure 1**. A total of 682 HCC patients who underwent liver resection in the First Affiliated Hospital of Xi’an Jiaotong University between January 2015 and December 2019 were included in this study. The inclusion criteria are listed as follows (1): patients with an accurate pathological diagnosis of HCC (2); patients who underwent radical hepatic resection and did not receive any preoperative antitumor therapies, such as trans-arterial chemoembolization (TACE), radiofrequency ablation, and antineoplastic drugs (3); patients with complete clinicopathologic, laboratory, imaging, and follow-up data. The exclusion criteria are listed as follows (1): the presence of perioperative death (2); the presence of other malignant tumors in the patient’s medical history (3); the presence of signs of macrovascular invasion before hepatectomy, such as portal vein invasion or hepatic vein invasion (4); patients who died without HCC recurrence within 2 years after surgery (5); the presence of extrahepatic metastasis within 2 years after surgery (6); patients who lost to the follow-up after surgery.

Finally, 481 patients were included. The included patients were assigned to the following two groups at a ratio of 7:3: the training cohort (n = 337) used to construct the model and the validation cohort (n = 144) used to assess the performance and verify the reliability of the model. (The randomized code was generated by the Random Number Generators of the SPSS statistical software). This retrospective study used anonymous data and was approved by the First Affiliated Hospital of Xi’an Jiaotong University.

### 2.2 Clinicopathologic and imaging data collection

Demographic data of patients included the patient’s age and sex. All patients received routine laboratory examinations before curative resection, such as blood tests, biochemical examinations, and tumor marker examinations. The research parameters in this study included serum AFP, aspartate aminotransferase (AST), alanine aminotransaminase (ALT), bilirubin, albumin, GGT, ALP, the count of neutrophils, platelets, and lymphocytes.

All pathological specimens were independently reassessed by two pathologists. The pathological sampling process was carried out in agreement with the practice guidelines for handling surgical specimens (23), and the sampling process included the sites, distances, and volumes for tissue sampling from HCC surgical specimens to make more accurate pathological diagnoses. Pathological features for the tumor included: tumor size, tumor differentiation, MVI grade, microscopic portal vein thrombus, intratumor necrosis, intratumor hemorrhage, bile duct invasion, capsular invasion, satellite nodules, and surgical margin. The typical pathological characteristics of HCC patients after surgery were shown in **Figures S1A–F**.

All patients received an upper abdominal contrast-enhanced CT scan preoperatively. Arterial-phase CT scans were obtained at 30 s, portal-phase CT scans were obtained at 60 s, and late-phase CT scans were obtained at 90–120 s. The scanning range and imaging parameters as described in our previous study (21). Peritumoral enhancement, peritumoral boundary, tumor shape, intratumoral artery, and multiple tumors in contrast-enhanced CT images were recorded and factored into the analysis. The typical imaging characteristics of HCC patients before surgery were shown in **Figures S1G–I**. The CT images were analyzed by two senior radiologists. In the case of inconsistency in assessment, they reached a consensus through reviewing and discussion, and the final analysis was based on their consensus.

### 2.3 Follow−up

Early HCC recurrence was defined as tumor recurrence within 2 years after liver resection. After tumor resection, all patients received regular follow-up according to clinical guidelines (24). Follow-up consisted of laboratory tests (e.g. serum AFP levels, liver function tests) and imaging examinations (ultrasound, CT, MRI) was performed every three months after surgery. A diagnosis of HCC recurrence was based on two or more examination methods including ultrasound, contrast-enhanced CT, MRI, or hepatic artery angiography with or without a raised serum AFP level. The typical imaging features of the recurrent HCC patient were shown in **Figure S2**. For patients without any evidence of recurrence, the last follow-up date was December 31, 2019.

### 2.4 Enzyme-linked immunosorbent assay

Whole blood samples from all enrolled patients were collected before hepatectomy and centrifuged at 3000 rpm for 10 min, and serum samples were immediately stored at -80°C until use. Serum concentrations of VEGF-A were measured with a VEGF ELISA Kit (R & D Systems China Co. Ltd., Shanghai, China), according to the manufacturer’s protocol. All ELISA assays were performed in duplicates.

### 2.5 Statistical analysis and development of nomogram model

Statistical analysis were performed with SPSS 27.0 software (IBM, New York, USA) and R software (version 3.4.4, R Project for Statistical Computing, http://www.r-project.org). Continuous variables were presented as means ± standard deviations (SD). Categorical variables were presented as percentages. The independent-sample t test was used for continuous variables. The Chi-squared test or Fisher’s exact test was used for categorical variables. X-tile software was applied to establish the optimal cutoff value of VEGF-A (25). The RFS rates within two years were calculated using the Kaplan–Meier method, and the log-rank test was used to compare differences among different groups. Univariate and multivariate analysis with Cox proportional hazards regression were used to determine the independent risk factors related to early recurrence in HCC patients in the training cohort. Variables that reached statistical significance (P < 0.05) in the univariate analysis were considered for the multivariate Cox regression model.

The nomogram for predicting 6-, 12-, and 24-month RFS was established based on proportionally converting each regression coefficient in multivariate Cox proportional hazards regression to a 0- to 100-point scale. The effect of the variable with the highest β coefficient (absolute value) was assigned 100 points. The points are added across independent variables to derive total points, which are converted to predicted probabilities. In addition, the best cut-off value for the total points was obtained using the X-tile software. Then, the enrolled patients were divided into low-, middle-, and high-risk subgroups to create a risk classification system, stratifying the recurrence risk of all HCC patients after tumor resection. The difference in RFS between the three subgroups was obtained using the Kaplan-Meier method. To quantify the predictive accuracy of the proposed nomogram, Harrell’s concordance index (C-index) was evaluated. The receiver operating characteristic (ROC) curves were constructed to estimate the performance of the model in the training cohort and internal validation cohort. All statistical tests were two-sided, and P < 0.05 indicated statistical difference.

## 3 Results

### 3.1 Comparison of the variables in the training and validation cohorts

A total of 682 HCC patients underwent liver resection in our center between January 2015 and December 2019. Finally, 481 patients were enrolled in this study according to the inclusion and exclusion criteria (**Figure 1**). The enrolled HCC patients were assigned to the following two groups at a ratio of 7:3: the training cohort (n = 337) and the validation cohort (n = 144).

The demographic, clinical, pathological, and imaging characteristics of HCC patients in the training cohort and validation cohort are shown in **Table 1**. The patients included 398 men and 83 women with an average age of 54.9 years, ranging from 15 to 79 years. Based on the follow-up data and diagnostic criteria, 182 (37.8%) patients experienced early tumor recurrence, while 299 (62.2%) patients had no recurrence evidence at the time of the last follow-up. 274 (54.9%) of patients were pathologically diagnosed as MVI-positive. 64% of HCC patients presented low AFP levels (AFP < 400 ng/mL). The mean serum VEGF-A concentrations in the training cohort and the validation cohort were 164.93 ± 100.66 pg/mL and 162.92 ± 100.57 pg/mL, respectively. There were no significant differences in the characteristics between the training and validation cohorts (**Table 1**).

**Table 1:** Demographic, clinical, pathological and imaging characteristics of HCC patients in the training and validation cohorts.

Variables	Overall cohort (n = 481)	Training cohort (n = 337)	Validation cohort (n = 144)	P-Value
Early recurrence				0.756
Absent	299 (62.2)	211 (62.6)	88 (61.1)	
Present	182 (37.8)	126 (37.4)	56 (38.9)	
Serum VEGF-A (pg/mL), mean ± SD	164.33 ± 100.53	164.93 ± 100.66	162.92 ± 100.57	0.841
Sex				0.626
Male	398 (82.7)	277 (82.2)	121 (84.0)	
Female	83 (17.3)	60 (17.8)	23 (16.0)	
Age (years)				0.540
≥ 60	160 (33.3)	115 (34.1)	45 (31.3)	
< 60	321 (66.7)	222 (65.9)	99 (68.8)	
Hepatitis				0.336
None	76 (15.8)	50 (14.8)	26 (18.1)	
HBV	387 (80.5)	272 (80.7)	115 (79.9)	
HCV	18 (3.7)	15 (4.5)	3 (2.1)	
Cirrhosis				0.076
Absent	118 (24.5)	75 (22.3)	43 (29.9)	
Present	363 (75.5)	262 (77.7)	101 (70.1)	
Diabetes				0.583
Absent	418 (86.9)	291 (86.4)	127 (88.2)	
Present	63 (13.1)	46 (13.6)	17 (11.8)	
AFP (ng/mL)				0.320
< 400	308 (64.0)	211 (62.6)	97 (67.4)	
≥ 400	173 (36.0)	126 (37.4)	47 (32.6	
ALT (U/L)				0.410
≤ 40	304 (63.2)	209 (62.0)	95 (66.0)	
> 40	177 (36.8)	128 (38.0)	49 (34.0)	
AST (U/L)				0.619
≤ 45	333 (69.2)	231 (68.5)	102 (70.8)	
> 45	148 (30.8)	106 (31.5)	42 (29.2)	
Total bilirubin (umol/L)				0.639
≤ 17.1	275 (57.2)	195 (57.9)	80 (55.6)	
> 17.1	206 (42.8)	142 (42.1)	64 (44.4)	
Direct bilirubin (umol/L)				0.106
≤ 3.4	131 (27.2)	99 (29.4)	32 (22.2)	
> 3.4	350 (72.8)	238 (70.6)	112 (77.8)	
GGT (U/L)				0.772
≤ 60	249 (51.8)	173 (51.3)	76 (52.8)	
> 60	232 (48.2)	164 (48.7)	68 (47.2)	
ALP (U/L)				0.383
≤ 125	358 (74.4)	247 (73.3)	111 (77.1)	
> 125	123 (25.6)	90 (26.7)	33 (22.9)	
Total protein (g/L)				0.584
≥ 65	238 (49.5)	164 (48.7)	74 (51.4)	
< 65	243 (50.5)	173 (51.3)	70 (48.6)	
Serum albumin (g/L)				0.695
≥ 40	184 (38.3)	127 (37.7)	57 (39.6)	
< 40	297 (61.7)	210 (62.3)	87 (60.4)	
Neutrophils (10^9^/L)				0.271
≤ 6.30	429 (89.2)	304 (90.2)	125 (86.8)	
> 6.30	52 (10.8)	33 (9.8)	19 (13.2)	
Platelet (10^9^/L)				0.799
≥ 125	273 (56.8)	190 (56.4)	83 (57.6)	
< 125	208 (43.2)	147 (43.6)	61 (42.4)	
Lymphocyte (10^9^/L)				0.225
≥ 1.10	274 (57.0)	198 (58.8)	76 (52.8)	
< 1.10	207 (43.0)	139 (41.2)	68 (47.2)	
Peritumoral enhancement				0.626
Absent	229 (47.6)	158 (46.9)	71 (49.3)	
Present	252 (52.4)	179 (53.1)	73 (50.7)	
Peritumoral boundary				0.821
Clear boundary	174 (36.2)	123 (36.5)	51 (35.4)	
Unclear boundary	307 (63.8)	214 (63.5)	93 (64.6)	
Tumor shape				0.948
Regular shape	186 (38.7)	130 (38.6)	56 (38.9)	
Irregular shape	295 (61.3)	207 (61.4)	88 (61.1)	
Intratumoral artery				0.199
Absent	242 (50.3)	176 (52.2)	66 (45.8)	
Present	239 (49.7)	161 (47.8)	78 (54.2)	
Multiple tumors				0.604
Absent	450 (93.6)	314 (93.2)	136 (94.4)	
Present	31 (6.4)	23 (6.8)	8 (5.6)	
Tumor size (mm)				0.835
≤ 50	237 (49.3)	165 (49.0)	72 (50.0)	
> 50	244 (50.7)	172 (51.0)	72 (50.0)	
Differentiation				0.759
Well	32 (6.7)	22 (6.5)	10 (6.9)	
Moderate	345 (71.7)	245 (72.7)	100 (69.5)	
Poor	104 (21.6)	70 (20.8)	34 (23.6)	
MVI grade				0.421
M0	217 (45.1)	148 (43.9)	69 (47.9)	
M1	112 (23.3)	84 (24.9)	28 (19.4)	
M2	152 (31.6)	105 (31.2)	47 (32.6)	
Microscopic portal vein thrombus				0.992
Absent	431 (89.6)	302 (89.6)	129 (89.6)	
Present	50 (10.4)	35 (10.4)	15 (10.4)	
Intratumor necrosis				0.516
Absent	382 (79.4)	265 (78.6)	117 (81.3)	
Present	99 (20.6)	72 (21.4)	27 (18.8)	
Intratumor hemorrhage				0.957
Absent	458 (95.2)	321 (95.3)	137 (95.1)	
Present	23 (4.8)	16 (4.7)	7 (4.9)	
Bile duct invasion				0.758
Absent	469 (97.6)	329 (97.6)	140 (97.2)	
Present	12 (2.5)	8 (2.4)	4 (2.8)	
Capsular invasion				0.372
Absent	144 (29.9)	105 (31.2)	39 (27.1)	
Present	337 (70.1)	232 (68.8)	105 (72.9)	
Satellite nodules				0.563
Absent	427 (88.8)	301 (89.3)	126 (87.5)	
Present	54 (11.2)	36 (10.7)	18 (12.5)	
Surgical margin (mm)				0.073
> 10.0	189 (39.3)	137 (40.7)	52 (36.1)	
5.0-10.0	168 (34.9)	107 (31.7)	61 (42.4)	
< 5.0	124 (25.8)	93 (27.6)	31 (21.5)	

### 3.2 Recurrence patterns of HCC patients

**Figure 2:**
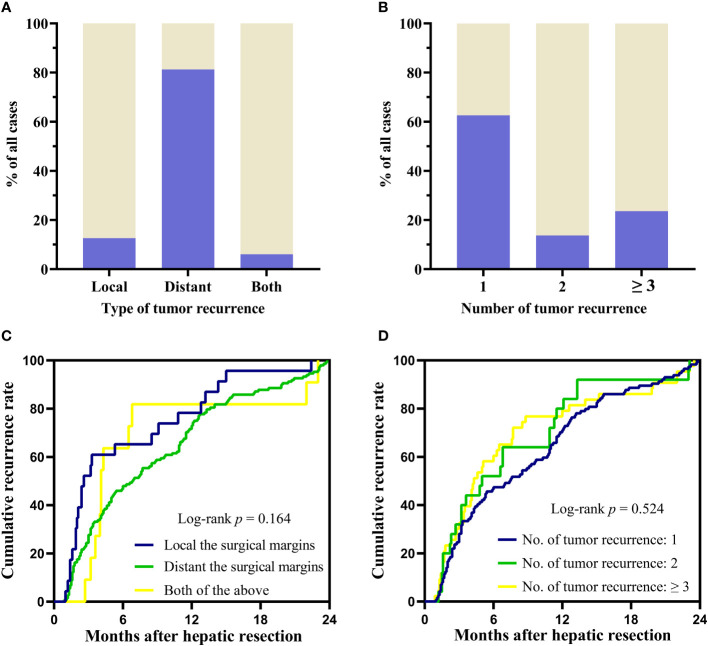
Recurrence patterns and differences between groups in TTR. **(A, B)** The pattern of recurrence according to the type and number of recurrent tumors. **(C, D)** Group differences in time to recurrence were examined with the log-rank test. TTR: time to recurrence.

The early recurrence patterns were assessed from three aspects (1): type of recurrent tumors: local recurrence at the surgical margins (recurrent tumors located within 20 mm of surgical margin), distant intrahepatic recurrence to the surgical margins (recurrent tumors located more than 20 mm of surgical margin) or both (2); number of recurrent tumors: solitary, two or ≥ 3 (3); time to recurrence (TTR) after surgery. The patterns of recurrences were shown in **Supplementary Table 1**. Among the 182 patients who developed tumor recurrence, 148 patients (81.32%) shown tumors distant to the surgical margin, 23 (12.64%) patients had recurrent tumors located within 20 mm of the surgical margin, the rest 11 patients had both distant and near margin recurrence (**Figure 2A**). 114 (62.64%) patients had solitary nodule. Whereas, 68 (37.36%) patients developed multinodular (2 or ≥3) recurrence (**Figure 2B**). Group differences in time to recurrence were examined with the log-rank test. There were no significant differences in TTR related to recurrence patterns (**Figures 2C, D**).

### 3.3 VEGF-A and MVI for predicting early recurrence

**Figure 3:**
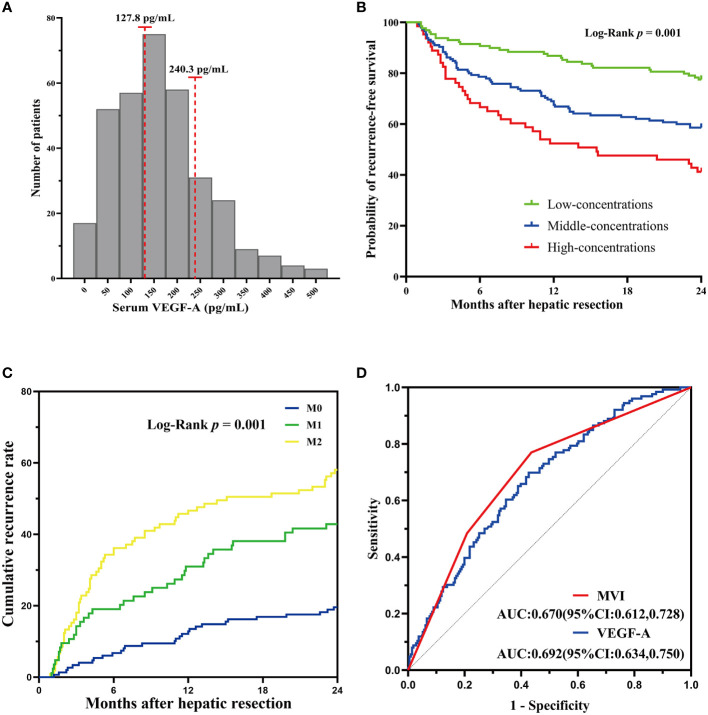
Serum VEGF-A and MVI correlated with HCC recurrence. **(A)**The cutoff value of VEGF-A concentrations was determined by the X-tile software. **(B)** Log-rank test for differences between low-, middle-, and high-concentrations groups shown significant differences. **(C)** The two-year cumulative recurrence rate among the three MVI subgrades. **(D)** ROC curves of MVI and VEGF-A for predicting early recurrence in HCC patients.

In that, VEGF-A was associated with MVI as shown in previous studies (21), we assessed VEGF-A expression in the present study. The patients in the training cohort were classified into the low-concentrations group (VEGF-A < 127.8 pg/mL, n = 181), middle-concentrations group (VEGF-A among 127.8 to 240.3 pg/mL, n = 145) and high-concentrations group (VEGF-A > 240.3 pg/mL, n = 63). The optimal cut-off values of VEGF-A for predicting RFS were determined by the X-tile bio-informatics software (**Figure 3A**). Log-rank test shown significant differences of RFS between low-, middle-, and high-concentrations groups (P = 0.001, **Figure 3B**). For MVI subgroups, the two-year cumulative recurrence rate in M0, M1, and M2 groups was 19.59%, 42.86%, and 58.1%, respectively (P < 0.001, **Figure 3C**). In order to determine the predictive ability of MVI and VEGF-A for early recurrence, the ROC curve was drawn by the survival ROC package of the R software. The areas under the ROC curve of MVI and VEGF-A in the training cohort were 0.670 (95%CI: 0.612-0.728) and 0.692 (95%CI: 0.634-0.750), respectively (**Figure 3D**). The result indicated MVI and VEGF-A had the good predictive ability for HCC recurrence.

### 3.4 Univariate and multivariate analysis of predictors of early recurrence

Univariate and multivariate Cox proportional hazards regression of risk factors for RFS were shown in **Table 2**. Factors, such as AFP ≥ 400 ng/mL, GGT > 60 U/L, VEGF-A among 127.8 to 240.3 pg/mL, VEGF-A > 240.3 pg/mL, peritumoral enhancement, unclear peritumoral boundary, irregular tumor shape, tumor size > 50 mm, MVI-positive, microscopic portal vein thrombus, intratumor necrosis, capsular invasion, surgical margin among 5.0 to 10.0 mm and surgical margin < 5.0 mm were found to be the possible predictors for recurrence of HCC by univariate analysis. By multivariable Cox regression analysis, AFP ≥ 400 ng/mL (P = 0.008), VEGF-A among 127.8 to 240.3 pg/mL (P = 0.012), VEGF-A > 240.3 pg/mL (P < 0.001), M1 subgroup of MVI (P = 0.002), M2 subgroup of MVI (P < 0.001), intratumor necrosis (P = 0.011), surgical margin among 5.0 to 10.0 mm (P = 0.043) and surgical margin < 5.0 mm (P = 0.012) were determined to be the independent risk factors associated with early recurrence.

**Table 2:** Cox proportional hazards regression analysis of risk factors for early recurrence in the training cohort.

Variables		Univariate analysis		Multivariate analysis	
		HR (95% CI)	P-Value	HR (95% CI)	P-Value
Sex	Male vs Female	1.174(0.728,1.894)	0.510		
Age (years)	≥ 60 vs < 60	1.145(0.787,1.666)	0.480		
Hepatitis	None		0.124		
	HBV vs None	1.464(0.853,2.513)	0.167		
	HCV vs None	0.579(0.168,2.001)	0.388		
Cirrhosis	Present vs Absent	1.464(0.924,2.319)	0.105		
Diabetes	Present vs Absent	1.186(0.736,1.913)	0.484		
AFP (ng/mL)	≥ 400 vs < 400	2.555(1.798,3.631)	< 0.001	1.662(1.144,2.415)	0.008
ALT (U/L)	> 40 vs ≤ 40	0.768(0.531,1.113)	0.164		
AST (U/L)	> 45 vs ≤ 45	1.104(0.761,1.603)	0.601		
Total bilirubin (umol/L)	> 17.1 vs ≤ 17.1	1.380(0.958,1.987)	0.083		
Direct bilirubin (umol/L)	> 3.4 vs ≤ 3.4	1.434(0.947,2.171)	0.089		
GGT (U/L)	> 60 vs ≤ 60	2.018(1.408,2.892)	< 0.001	—	0.126
ALP (U/L)	> 125 vs ≤ 125	1.324(0.910,1.927)	0.142		
Total protein (g/L)	< 65 vs ≥ 65	0.743(0.523,1.055)	0.097		
Serum albumin (g/L)	< 40 vs ≥ 40	0.889(0.623,1.269)	0.517		
Neutrophils (10^9^/L)	> 6.30 vs ≤ 6.30	1.307(0.685,2.493)	0.417		
Platelet (10^9^/L)	< 125 vs ≥ 125	1.207(0.847,1.721)	0.298		
Lymphocyte (10^9^/L)	< 1.10 vs ≥ 1.10	1.126(0.787,1.611)	0.516		
VEGF-A (pg/mL)	< 127.8		< 0.001		0.001
	127.8-240.3 vs < 127.8	2.134(1.369,3.325)	0.001	1.781(1.135,2.797)	0.012
	> 240.3 vs < 127.8	3.495(2.147,5.690)	< 0.001	2.552(1.527,4.266)	< 0.001
Peritumoral enhancement	Present vs Absent	1.499(1.047,2.145)	0.027		0.422
Peritumoral boundary	Unclear boundary vs Clear boundary	2.091(1.393,3.137)	< 0.001	—	0.160
Tumor shape	Regular shape vs Irregular shape	1.601(1.097,2.337)	0.015	—	0.703
Intratumoral artery	Present vs Absent	1.356(0.956,1.925)	0.088		
Multiple tumors	Present vs Absent	0.974(0.494,1.919)	0.939		
Tumor size (mm)	> 50 vs ≤ 50	1.703(1.192,2.434)	0.003	—	0.610
Differentiation	Well		0.397		
	Moderate vs Well	1.863(0.758,4.578)	0.175		
	Poor vs Well	1.845(0.708,4.804)	0.210		
MVI grade	M0		< 0.001		< 0.001
	M1 vs M0	2.599(1.593,4.240)	< 0.001	2.221(1.349,3.656)	0.002
	M2 vs M0	4.092(2.627,6.374)	< 0.001	3.120(1.977,4.925)	< 0.001
Microscopic portal vein thrombus	Present vs Absent	1.759(1.079,2.867)	0.023	—	0.584
Intratumor necrosis	Present vs Absent	1.913(1.307,2.799)	0.001	1.666(1.126,2.464)	0.011
Intratumor hemorrhage	Present vs Absent	1.324(0.618,2.838)	0.471		
Bile duct invasion	Present vs Absent	0.68(0.169,2.756)	0.591		
Capsular invasion	Present vs Absent	1.513(1.009,2.270)	0.045	—	0.267
Satellite nodules	Present vs Absent	1.620(0.984,2.670)	0.058		
Surgical margin (mm)	> 10.0		< 0.001		0.027
	5-10 vs > 10.0	1.959(1.262,3.041)	0.003	1.601(1.016,2.525)	0.043
	< 5.0 vs > 10.0	2.248(1.445,3.498)	< 0.001	1.790(1.139,2.812)	0.012

**Figure 4:**
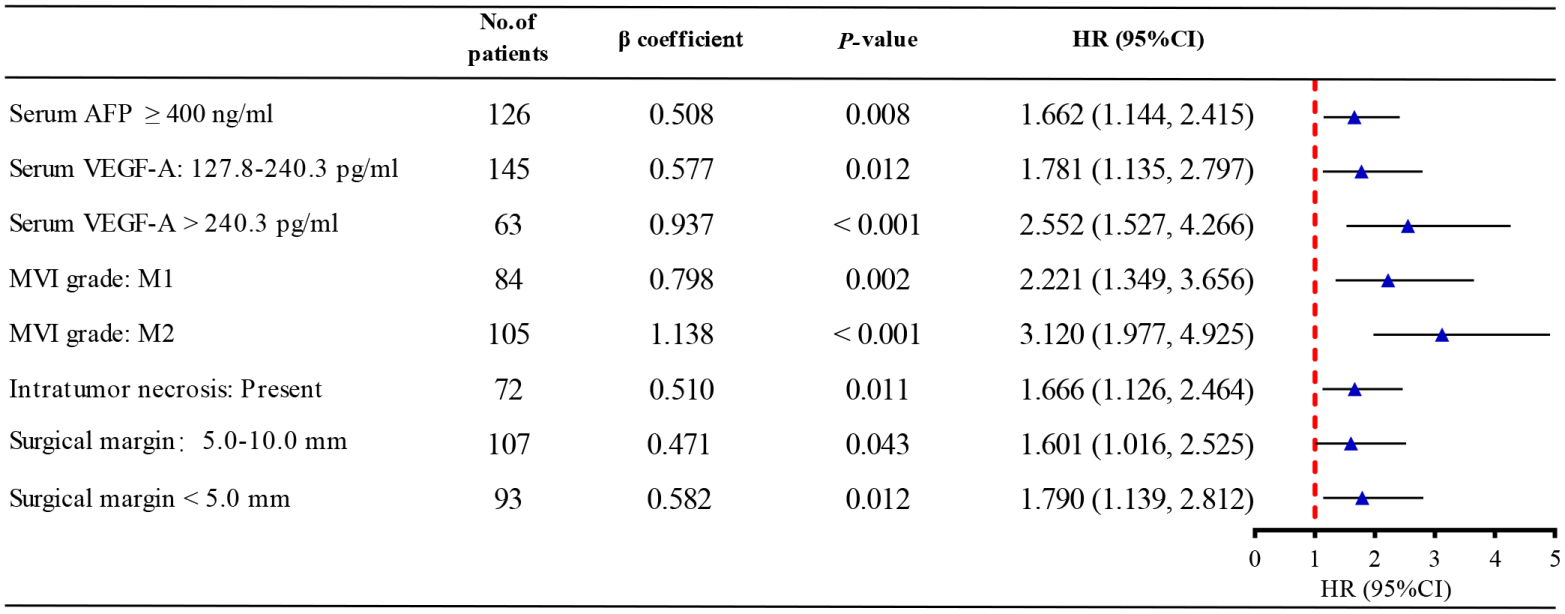
The forest plot of multivariate Cox regression analysis. AFP, alpha fetoprotein; VEGF-A, Vascular endothelial growth factor A; MVI, microvascular invasion; HR, hazard ratio.

A Forest plot was drawn to show the effect of different risk factors on patient RFS (**Figure 4**). M2 subgroup of MVI had the strongest predictive power for early recurrence (HR: 3.120; 95%CI: 1.977-4.925).

### 3.5 Development and validation of the nomogram model

**Figure 5:**
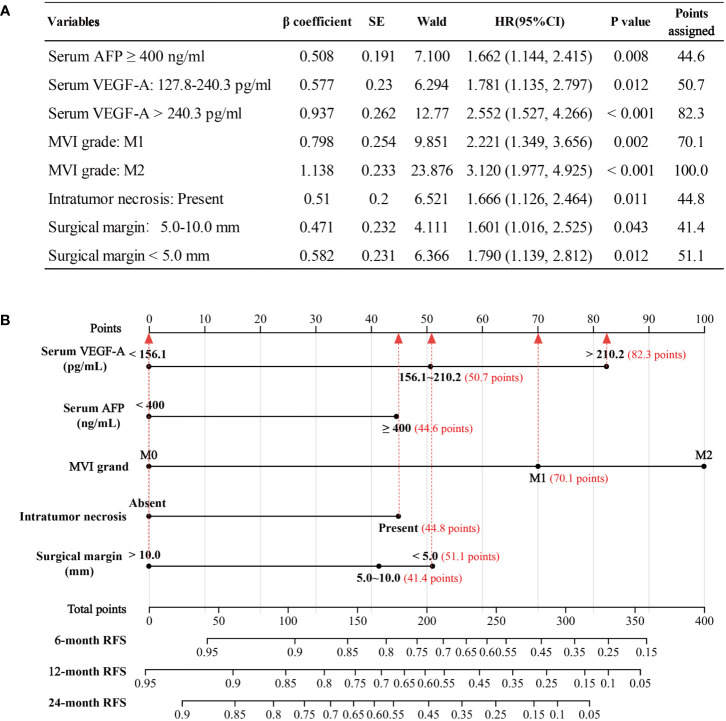
Development of the nomogram model. **(A)** The points assignments of variables based on Cox regression analysis. **(B)** Nomogram model depicting 6−, 12− and 24−month RFS. The use of nomogram was as follows: Points are assigned for each variable by drawing a straight line upward from the corresponding value to the “Points” line. Then, sum the points received for each variable, and locate the number on the “Total Points” axis. The 6−, 12− and 24−month RFS can be calculated by connecting each point to the survival line. AFP, alpha fetoprotein; VEGF-A, Vascular endothelial growth factor A; MVI, microvascular invasion, RFS, recurrence−free survival.

The nomogram was based on proportionally converting each regression coefficient in multivariate Cox regression analysis to a 0- to 100- point scale. The effect of the variable with the highest β coefficient was assigned 100 points (**Figure 5A**). A nomogram was developed to predict 6−, 12− and 24−month RFS for patients with HCC after radical hepatic resection based on the significant risk factors identified in the Cox regression analysis (**Figure 5B**). The M2 subgroup of MVI had the highest score (100 points). The scores for the other variables were: 44.6 points (serum AFP ≥ 400 ng/mL), 50.7 points (serum VEGF-A among 127.8 to 240.3 pg/mL), 82.3 points (serum VEGF-A > 240.3 pg/mL), 70.1 points (M1 subgroup of MVI), 44.8 points (intratumor necrosis), 41.4 points (surgical margin among 5.0 to 10.0 mm), and 51.1 points (surgical margin of < 5.0 mm).

**Figure 6:**
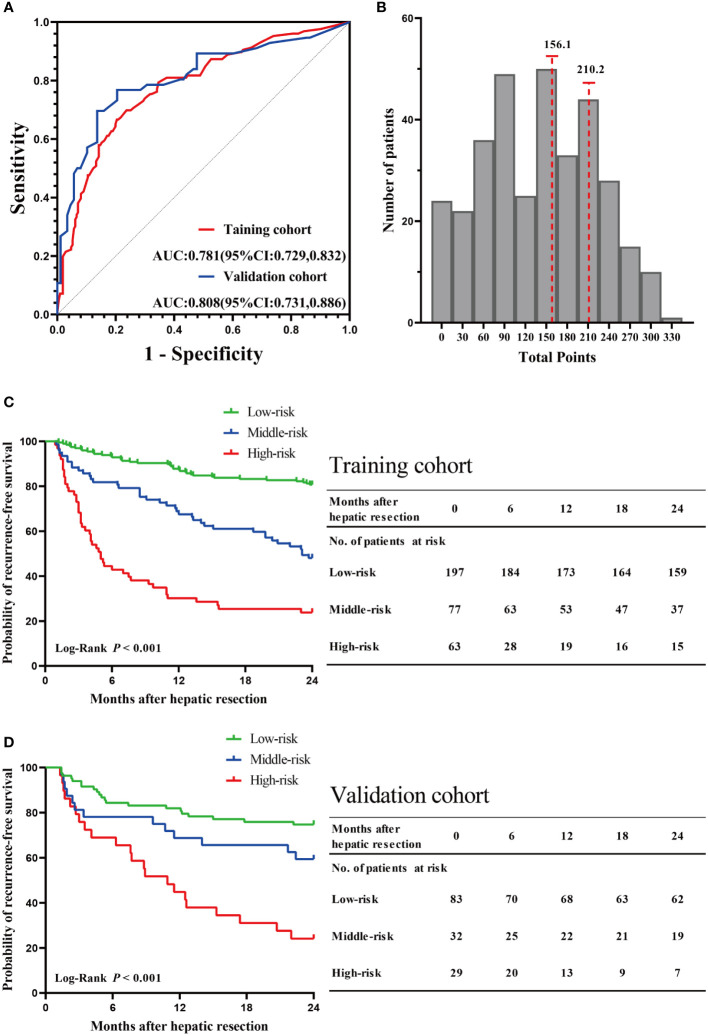
Validation of the nomogram model. **(A)** The ROC curve and AUC with 95% CI in the training cohort and validation cohort. **(B)** The two optimal cutoff points of the risk scores were determined by the X-tile software. Kaplan–Meier survival analysis of the high-risk, middle-risk, and low-risk HCC patients in the training cohort **(C)** and validation cohort **(D)**.

The C-indexes for RFS prediction were 0.744 (95%CI: 0.701-0.787) and 0.736 (95%CI: 0.672-0.801) for the training and validation cohorts, respectively. To evaluate and compare the discriminatory power of the nomogram model, we plotted the ROC curve and calculated the area under the ROC curve (AUC) with 95% confidence intervals (CI) in the training cohort and validation cohort, respectively. The area under the ROC curve of the established nomogram in the training cohort and validation cohort was 0.781 (95% CI: 0.729-0.832) and 0.808 (95% CI: 0.731-0.886), respectively (**Figure 6A**). At the individual level, the risk scores were calculated for each patient based on the developed risk model. According to the risk scores of patients, we acquired two optimal cutoff points using the X-tile software: 156.1 and 210.2 (**Figure 6B**). Then, we obtained the RFS rates of low-, middle-, and high-risk groups were 80.71% (159 of 197), 48.05% (37 of 77), and 23.81% (15 of 63), respectively. Intergroup comparison shown a statistically significant difference in 2-year RFS using Kaplan–Meier survival analysis in the training cohort (P < 0.001, **Figure 6C**). Likewise, consistent findings were also presented in the validation cohort (P < 0.001, **Figure 6D**).

## 4 Discussion

Tumor recurrence is one of the most significant poor prognostic factors for patients with HCC (26). In this retrospective study, early recurrence occurred in 37.8% of the 481 patients who underwent curative liver resection of HCC. Most recurrences (133 of 182 [73.08%]) occurred within 1 year after surgery. Microvascular invasion, elevated serum concentrations of VEGF-A and AFP, intratumor necrosis, and surgical margin were independent risk factors of early recurrence. By incorporating these risk factors, we developed and validated a multivariable nomogram model to predict the 6-, 12-, and 24-month RFS of HCC patients after R0 resection.

Microvascular invasion is tumor emboli in vascular cavity with adhesion to endothelial cell observed under a microscope, which mainly occurs in portal vein (27). Residual tumor cells or tumor emboli spreading *via* the portal vein conserve elements of their primary tumor tissue after surgery. The tumor emboli easily form metastatic foci in the liver, exhibiting cancer recurrence subsequently (28). Moreover, the presence of MVI can change the pathophysiological environment in liver and promote the spreading of cancer cells. This change enhances angiogenesis, the ability of the tumor to induce new vessel formation in order to grow (29). Another plausible mechanism to achieve early recurrence is that microvascular invasion occurs when HCC cells have developed a sufficiently evolved phenotype to invade blood vessels, such as mutations in oncogenes and tumor suppressor genes with activation of several signaling pathways (30).

Tumor angiogenesis is one important stage of microvascular invasion and the hallmark of tumor progression (31). The angiogenesis-related growth factors, such as VEGF-A, could trigger biological effects including endothelial proliferation and migration, and the formation and branching of new tumor blood vessels necessary for rapid tumor growth and recurrence (32). The newly formed vessels in tumor have abnormally leaky vasculature, partially due to the overexpression of VEGF, resulting in areas of high interstitial pressure and severe hypoxia or necrosis, both of which can further drive malignant potential (33). Since a significantly increased expression of VEGF-A was found in HCC patients with early recurrence, we incorporated VEGF-A into our newly-built nomogram model.

Regions within solid tumors often experience mild to severe oxygen deprivation, due to aberrant vascular function (34). The rapid growth of large tumors may lead to an increase in tumor vascularization and form a hypoxic microenvironment, which induces tumor necrosis (35, 36). Although angiogenesis is stimulated by increased expression of VEGF-A, neovascularization has abnormal structure and function, leading to impaired perfusion, eventually leading to intratumor necrosis (37). The chronic intratumor necrosis in the hypoxic region fuel tumor progression (38). Moreover, tumors with intratumor necrosis are more likely to be invasive and to recur through suppression of antitumor immunity (39).

In this study, AFP was a risk factor of tumor recurrence after curative liver resection of HCC. Elevated AFP level is often used as a serological marker for HCC and strongly correlates with the invasiveness of cancer cells (40). AFP promotes tumor growth and recurrence *via* immunosuppression and tumor vascularization (41, 42).

A wider resection margin (e.g. ≥ 1 cm) is recommended strategy in the clinical guideline (2) in order to reduce the risk of positive surgical margin and tumor recurrence. Despite extensive studies, the optimal liver resection margin is still controversial. In present study, HCC patients with a surgical margin < 5.0 mm show a significantly higher incidence of tumor recurrence (HR:1.790, 95%CI:1.139-2.812; P =0.012). Hence, for those patients with very narrow-margin resection (< 5 mm), preventative intervention (i.e., adjuvant therapies such as TACE or radiotherapy) should start at the earliest opportunity.

By combining blood biomarkers and pathological parameters, we established a multivariable nomogram to predict the 6-, 12-, and 24-month RFS of HCC. The nomogram indicated good predictive performance with an AUC of 0.781 (95% CI: 0.729-0.832) and 0.808 (95% CI: 0.731-0.886) in the training and validation cohorts, respectively. One of the greatest advantages of the nomogram model in the present study was that all the parameters incorporated were easily acquired so that the model could be easily applied in clinical practice. The model could help identify patients at a high risk of recurrence and call for closer and more stringent recurrence surveillance for these patients. However, the present study has limitations. First, this is a single-center retrospective study, which may has selection bias. Second, further data from external validation cohort are needed to make the results more convincing.

In conclusion, elevated serum concentrations of AFP and VEGF-A, microvascular invasion, intratumor necrosis, and surgical margin were independent risk factors of early recurrence. A multivariable nomogram model was developed and validated, which could quantify the risk of recurrence after R0 resection for HCC patients, and shown desirable effectiveness in predicting 6-, 12-, and 24-month recurrence-free survival.

## Data availability statement

The data analyzed in this study is subject to the following licenses/restrictions: The raw data supporting the conclusions of this article are available from the corresponding authors on reasonable request. Requests to access these datasets should be directed to hanshaoshan@xjtufh.edu.cn.

## Ethics statement

The protocol was reviewed and approved by the Institutional Review Board (IRB) of the First Affiliated Hospital of Xi’an Jiaotong University with waiver for informed consent (No. XJTU1AF2022LSK-431).

## Author contributions

HW, QL and SH conceived and designed of the study. QL supervised the study. QL and SH obtained the research fund. HW and SH screened the publications, performed statistics, and drafted the manuscript. RK-L and HM helped perform statistics. HM and RT-L screened the publications. JL offered insight and guidance on histopathological analysis. All authors contributed to the article and approved the submitted version.

## Supplementary material

Supplementary Figure 1 Postoperative pathological parameters and preoperative radiographic parameters. **(A)** M0; **(B)** M1; **(C)** M2; **(D)** Intratumor necrosis; **(E)** Capsular invasion; **(F)** Surgical margin ≤ 5.0 mm;**(G)** Peritumoral enhancement; **(H)** Unclear peritumoral boundary; **(I)** Multiple tumors.

Supplementary Figure 2 Diagnostic legends of HCC recurrent patients. HCC recurrence was confirmed using contrast-enhanced CT **(A)**, MRI **(B)**, and hepatic artery angiography **(C, D)**.
