# Supplementary figures and images for: A novel nomogram predicting the early recurrence of hepatocellular carcinoma patients after R0 resection
Source: Front Oncol. 2023 Mar 17;13:1133807. doi: 10.3389/fonc.2023.1133807 (PMC10063973; doi:10.3389/fonc.2023.1133807)

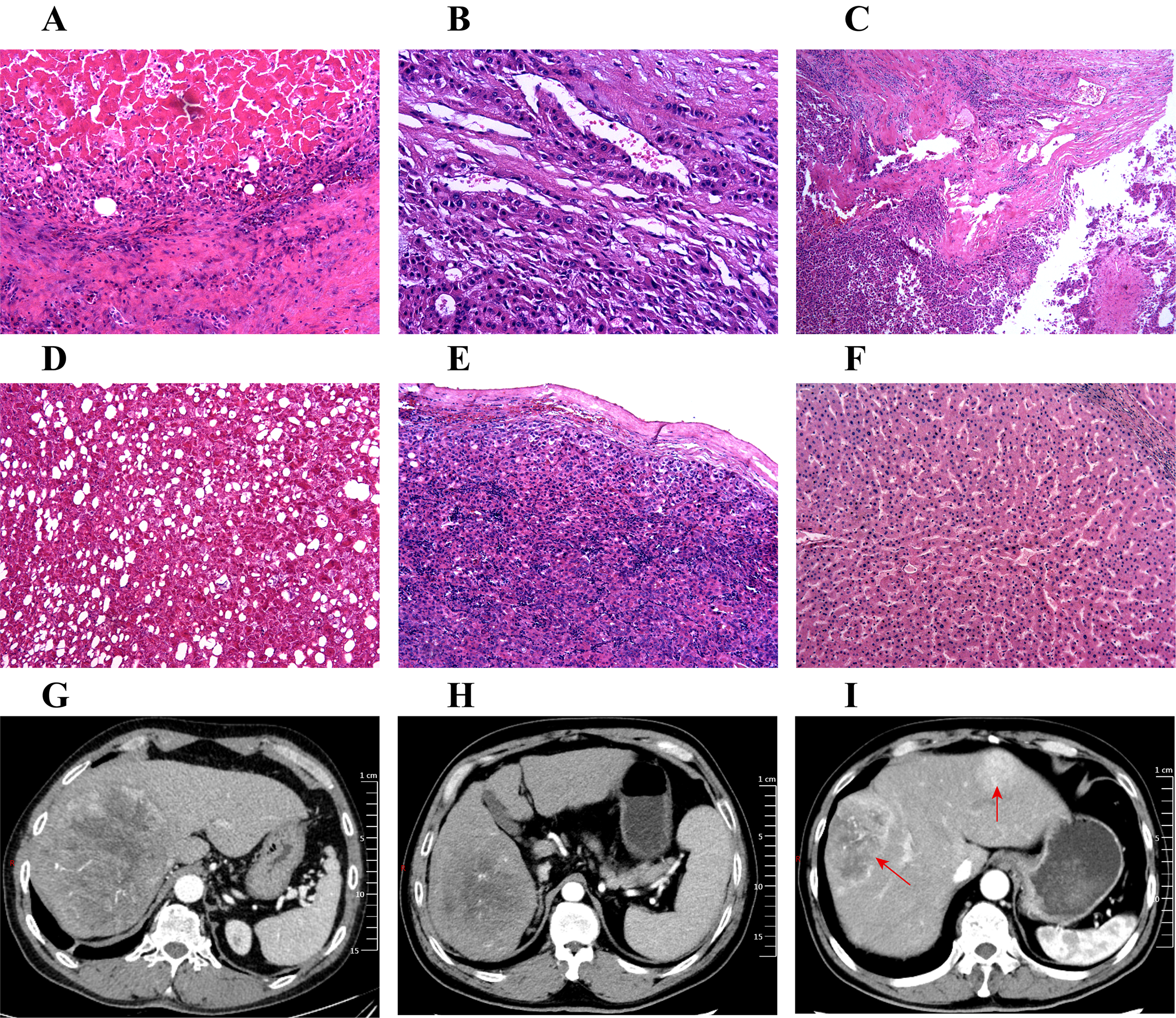

Supplement: Supplementary Figure 1 — Postoperative pathological parameters and preoperative radiographic parameters. (A) M0; (B) M1; (C) M2; (D) Intratumor necrosis; (E) Capsular invasion; (F) Surgical margin ≤ 5.0 mm;(G) Peritumoral enhancement; (H) Unclear peritumoral boundary; (I) Multiple tumors. [file Image_1.tif]

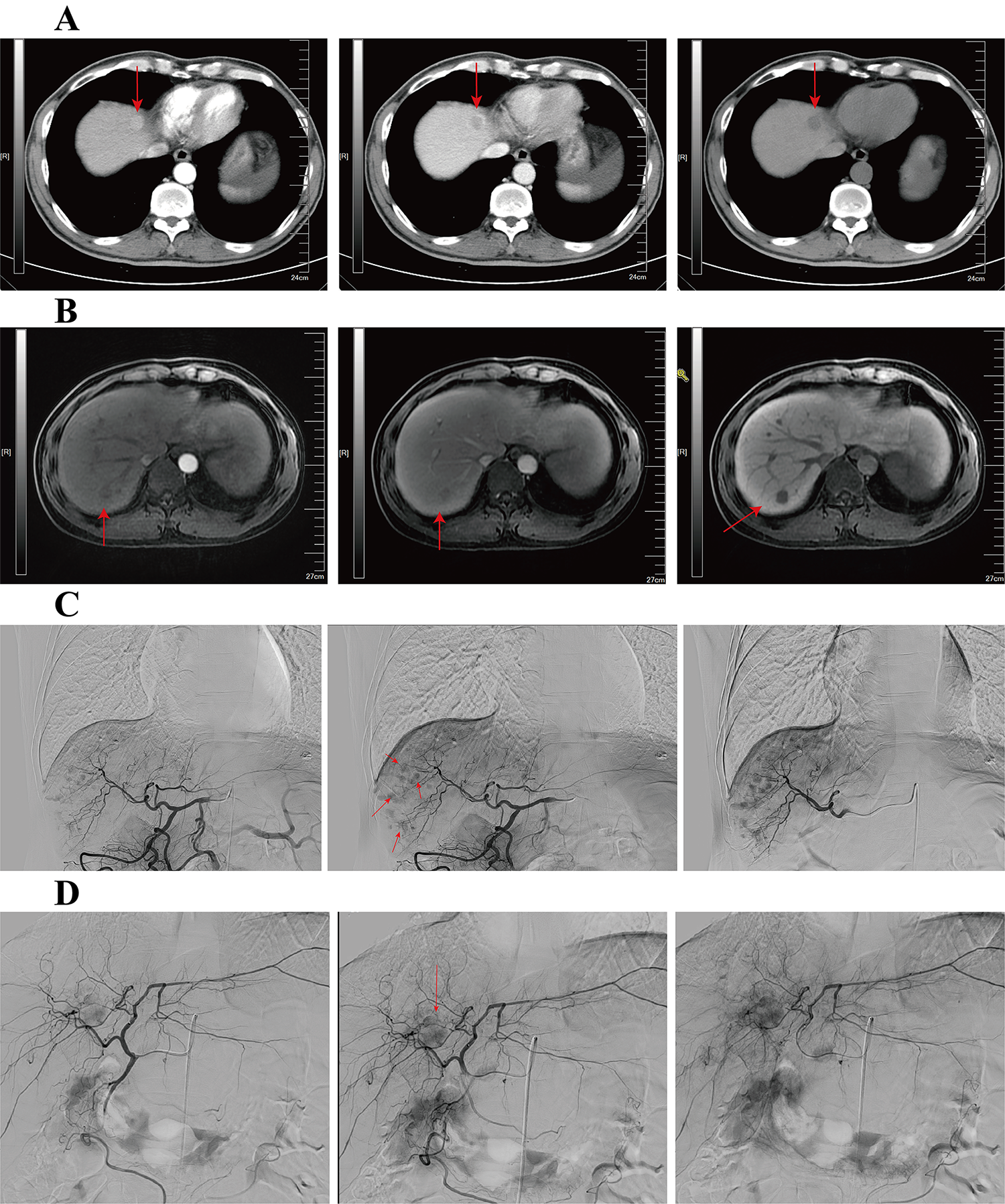

Supplement: Supplementary Figure 2 — Diagnostic legends of HCC recurrent patients. HCC recurrence was confirmed using contrast-enhanced CT (A), MRI (B), and hepatic artery angiography (C, D). [file Image_2.tif]
