# Supplementary material for: A novel nomogram predicting the early recurrence of hepatocellular carcinoma patients after R0 resection
Source: Front Oncol. 2023 Mar 17;13:1133807. doi: 10.3389/fonc.2023.1133807 (PMC10063973; doi:10.3389/fonc.2023.1133807)
Supplement: Supplementary file 3 [file Table_1.docx]

Supplementary Material

A novel nomogram predicting the early recurrence of hepatocellular carcinoma patients after R0 resection

Huanhuan Wang^1^, Runkun Liu^1^, Huanye Mo^1^, Runtian Li^1^, Jie Lian^2^, Qingguang Liu^1^*, Shaoshan Han^1^*

*** Correspondence:**Qingguang Liu：liuqingguang@vip.sina.com; Shaoshan Han：hanshaoshan@xjtufh.edu.cn

# Supplementary Table 1 Patterns of recurrences in HCC patients

|  | **No. of patients** | **Median** **TTR and 95%CI (months)** | **P-Value** |
| --- | --- | --- | --- |
| Type of tumor recurrence |  |  | 0.164 |
| Local recurrence | 23(12.64) | 2.60(1.31-.3.90) |  |
| Distant intrahepatic recurrence | 148(81.32) | 6.80(4.94-8.66) |  |
| Both of the above | 11(6.04) | 4.10(3.78-4.42) |  |
| Number of tumor recurrence |  |  | 0.524 |
| 1 | 114(62.64) | 7.40(4.26-10.54) |  |
| 2 | 25(13.73) | 5.00(0.10-9.90) |  |
| ≥3 | 43(23.63) | 4.30(3.23-5.37) |  |

# P-value was calculated by log-rank test to compare the differences in the time to recurrence between each subgroup. TTR, time to

# recurrence.
